# Help Wanted, Experience Preferred, Stamina a Must: A Narrative Review of the Contextual Factors Influencing Nursing Recruitment and Retention in Rural and Remote Western Canada from the Early Twentieth Century to 2023

**DOI:** 10.1177/08445621231204962

**Published:** 2023-10-06

**Authors:** Amanda M. McCallum, Helen E. R. Vandenberg, Kelly L. Penz

**Affiliations:** 1 70397 University of Saskatchewan College of Nursing, Saskatoon, SK, Canada

**Keywords:** Rural and remote nursing, recruitment and retention, Western Canada, history, nursing, twentieth century, narrative review

## Abstract

Rural and remote communities of Western Canada have struggled to recruit and retain nursing professionals since the turn of the twentieth century. Existing literature has identified the unique challenges of rural nursing due to the shifting context of rural and remote nursing practice. The objective of this narrative review is to explore the history of rural and remote nursing to better understand the contextual influences shaping rural nursing shortages in Western Canada. This narrative review compared 27 sources of scholarly and historical evidence on the nature of rural nursing practices and recruitment and retention methods following the First World War until 2023. The findings suggest that the complex nature of rural nursing practice is a consistent challenge that has intersected with the long-standing power inequities that are inherent in rural marginalization, political influences, the nursing profession, social structures, and organizational design, to perpetuate rural nursing shortages throughout the past century. Integration and collaboration are needed to reduce systemic marginalization and develop effective and sustainable solutions to reduce nursing shortages in rural and remote areas of Western Canada.

Nurses have played a fundamental role in the development and delivery of rural and remote healthcare in Western Canada (Jones et al., 2019). In the nursing literature, “rural” is often defined as a community with a population of fewer than 10,000 people, where less than half of the employed population commutes to urban centers for work (MacLeod et al., 2017). Canada's vast landscape creates challenges for equitable healthcare delivery, especially in sparsely populated regions (Tipliski, 2004). It is predominantly nurses who are responsible for the coordination and delivery of rural and remote healthcare services (Martin-Misener et al., 2020). Yet, rural and remote regions encounter ongoing struggles to recruit and retain adequate levels of nursing staff to support public health and acute care needs of the communities (Kulig et al., 2015). Few contemporary scholars have attempted to understand why nursing recruitment and retention remains a significant problem in rural Canada (Ariste, 2019; Kulig et al., 2015).

Nursing historians have long suggested that contemporary scholars need to better capitalize on the knowledge and innovation of historical literature (Elliot, 2010). Nursing historian Grypma (2017) argues that “knowing how nurses have successfully responded to professional and practice concerns in the past can provide nurses with strategies and confidence to try new (old) clinical interventions or to interrupt concerning social or political trajectories” (p. 9). Learning about nursing history supports innovation, challenges current thinking, offers context for current practice, and reveals critical explanations of nursing's roles within society (Grypma, 2017). The purpose of this narrative review is to explore the history of rural and remote nursing to better understand the contextual influences shaping rural nursing shortages in Western Canada.

## Background

Nursing historians often suggest that inadequate nurse staffing in rural and remote regions has been a challenge since the dawn of professional nursing (Elliot, 2010; Quiney, 2008). Following WWI, emerging threats to individual health, combined with social and political influences, spurred a sharp increase in the demand for healthcare services (Quiney, 2008). A variety of missionaries and the Canadian Red Cross Society (CRCS) developed hospitals and nursing outposts across many Canadian provinces to bring health and “civilization” to rural and remote communities that were disadvantaged by social, economic, and health-related factors (Elliott, 2008; Quiney, 2008). This was long before the development of multicultural policy in Canada during the 1980s, when immigrants and Indigenous populations were expected to assimilate to the Anglo-Saxon Christian ideal (Vandenberg & Gallagher-Cohoon, 2021). The CRCS struggled to find suitable candidates who possessed the nursing skills, confidence, stamina, and grit to not only fill, but also succeed amongst the physical, mental, environmental, and social demands of rural and remote regions (Quiney, 2008).

Fast forward to the twenty-first century, and several social and contextual factors have shifted, but nursing shortages continue to be a global phenomenon further impeded by the geographical imbalance of urban and rural settings (MacLeod et al., 2017). Rural residents are often underserviced in health and social services, due to the insufficient numbers of accessible healthcare professionals (Rohatinsky et al., 2020). Governments expect the shortages of healthcare professionals to worsen due to an aging workforce and the increased demands associated with growing rates of chronic diseases (Government of Northwest Territories (GNWT), 2022). Nursing shortages are compounded by increased stress and burnout among existing staff who face unmanageable workload demands and a continued lack of understanding from decision-makers regarding the complexity of nursing practice (GNWT, 2022; Tallman & Bruning, 2005). The 2019 emergence of the novel coronavirus (SARS-CoV2 or COVID-19) and the subsequent pandemic have worsened the problem further (Lopez et al., 2022).

In Canada, provincial political leaders and publicly funded healthcare authorities administer and deliver health services within each province. Western Canada has historically been influenced by a shift from more socialist and welfarism-minded governments in the post-World War II era to a neoliberal political ideology since the 1970s (Foth & Holmes, 2017; Richardson, 2001). The shifting political structures in Canada directly influenced nursing's image, status, and power throughout the twentieth century, most often by ideologically opposing the advancement of nursing from a trade to a profession (Elliott, 2008; Richardson, 2001; Tipliski, 2004). Nursing scholars have further suggested that the lack of development in nursing has been influenced by patriarchal social systems, which prevent the development of female-dominated professions (Ashley, 1976; Elliott, 2008). The lack of support for rural nursing practice, and nursing in general, likely influences the current situation. A review of historical and contemporary literature is needed to better understand the contextual factors shaping rural nursing shortages in Western Canada.

## Method

A narrative review method was utilized for this study to consider a more extensive range of time, sources, and the synthesis of knowledge deemed valuable in presenting a particular topic or point of view (Cronin et al., 2008; Pare & Kitsiou, 2017). Although historical nursing scholarship often does not meet the methodological requirements of typical systematic literature review designs, it provides the opportunity to explore the context related to this topic.

### Selecting the topic

We focused the review on the Western Canadian provinces of British Columbia, Alberta, Saskatchewan, and Manitoba to limit the scope of the review to a key geographical and sociopolitical area. This review is focused primarily from post-WWI to present to capture the rapid growth of modern healthcare organizations and the movement toward standardization of hospital and nursing care that continue to influence Western Canadian healthcare today (Vandenberg & Johnson, 2022). Much of the historical literature focuses on the mid-twentieth century when outpost nursing was first established.

### Searching and retrieval of the literature

**Figure 1.**
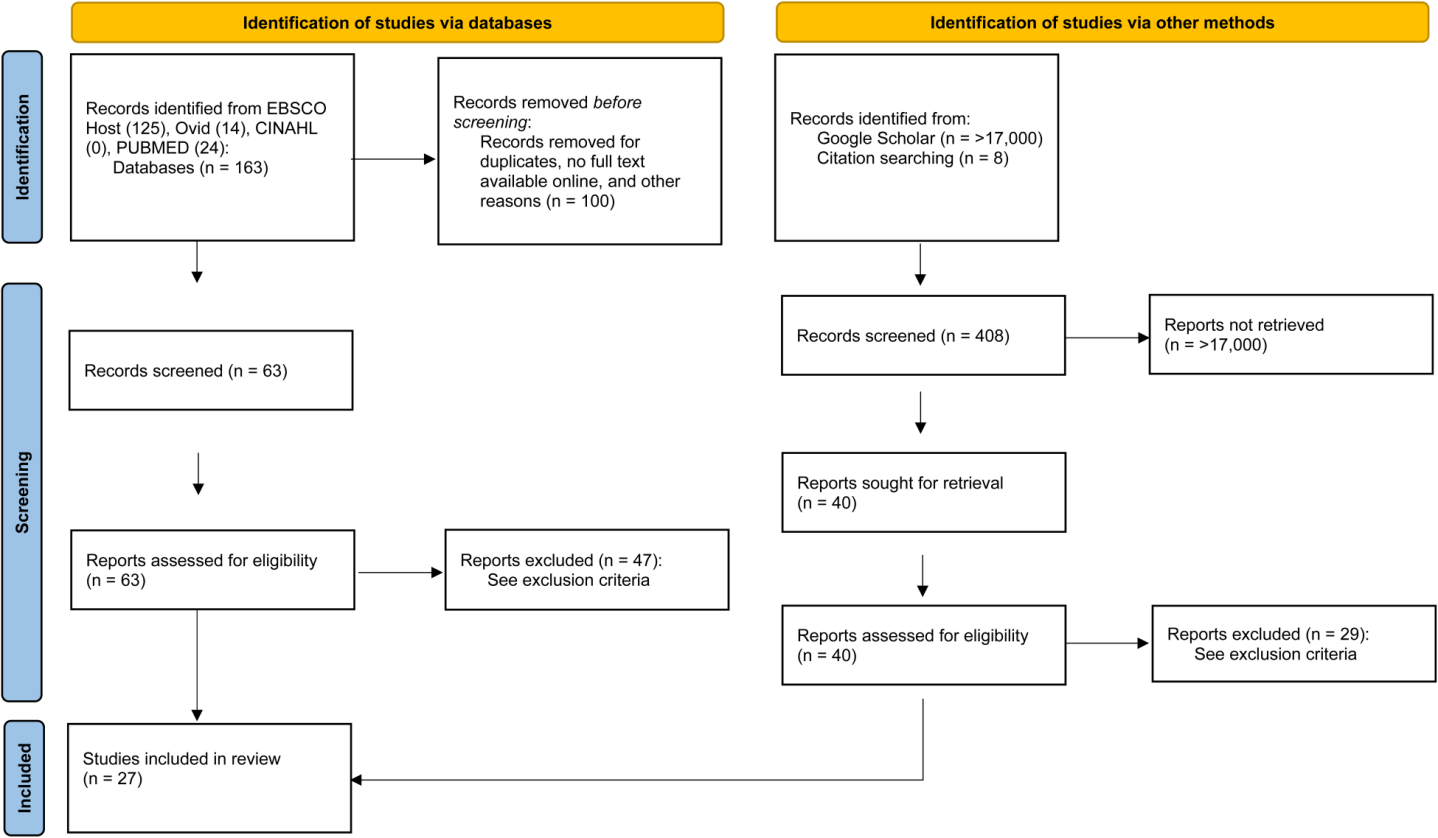
PRISMA flow diagram (Page et al., 2021). Note: Exclusion criteria: target population other than nursing, focused on Eastern Canadian context without reference to Western Canada, focused on urban context. Inclusion criteria: written in English, full-text available online or in accessible print, specific to the nursing profession, focused on Canadian or Western Canadian context, include information regarding the nature of rural and remote nursing practice and recruitment and retention of nursing personnel at a point in time between 1918 and 2023.

A PRISMA diagram outlines the search strategy for this study (Figure 1). Consistent with Cronin et al.'s (2008) narrative review approach we included the following databases: EBSCO Host, Ovid, CINAHL, and Pub Med. The Ovid, CINAHL, and Pub Med databases were chosen because of their vast indexing of literature spanning the field of nursing and multidisciplinary healthcare systems. EBSCO Host offered a potential source for digitally available books, government reports, and historical content. Inclusion criteria included content that was written in English, available online or in accessible printed text, specific to the nursing profession, focused on Canadian or Western Canadian context, and included information regarding the nature of rural and remote nursing practice and recruitment and retention of personnel at a point in time between 1918 and 2023. Database searches returned 63 articles for screening, with 16 articles meeting inclusion criteria and selected for review.

Due to the scarcity of articles focusing on the historical scholarship of rural and remote nursing, the Google Scholar database was also utilized, resulting in 17,000 new entries. As per the recommendations of Haddaway et al. (2015), the first 200 articles of each were screened for inclusion until a point of topic saturation. Content from backward searches and seminal literature was added. The search returned 40 articles for retrieval, with 11 articles meeting inclusion criteria and selected for review.

## Findings

The 27 studies selected for review were categorized as being historical (n = 13), examining rural nursing between 1920 and 1999, and contemporary (n = 14), examining rural nursing between 2000 to 2023. The literature was first reviewed to understand similarities and differences in the context of rural and remote nursing during the historical and contemporary time periods. The contextual overview is critical to this review to understand what historical influences have changed, and what remains very relevant to discussions of rural nursing shortages. Next, the literature was organized into a table summarizing key factors and recommendations for practice related to the recruitment and retention of nurses in rural and remote locations (see Appendix A). The analysis revealed five primary themes or key factors that have contributed to nursing recruitment and retention challenges in rural regions throughout the historical and contemporary periods. The five themes identified in the analysis include: The complexity of rural and remote nursing practice; Strategies to support increased recruitment and retention of rural and remote nurses; The impact of rural marginalization and political influences; and The impact of an urban majority mindset and subsequent power imbalances.

### The contexts of rural and remote nursing in the twentieth and twenty-first century

During the 1930s to 1950s, nurses were often deployed to outpost nursing stations by governments to improve the health outcomes of remote Indigenous communities and rural immigrant populations (McBain, 2012). Nurses working in these stations were uneducated about Indigenous languages and cultures and lacked the equipment, transportation, communication systems, and basic amenities like clean tap water that was available for nurses in Southern regions (Drees & McBain, 2001; McBain, 2012). Southern societies considered the rural North to be a masculine, rough, and dangerous environment (Elliott, 2008; Rutherdale, 2010). Qualities deemed to be valuable to outpost nurses were independence, confidence, adventurousness, challenge-seeking, and a desire for autonomy (Elliott, 2008). The nature of rural and remote nursing practice and lifestyle was a stark departure from the social norms for women in urban society during that time. During the early to mid-twentieth century, female workforce participation rates were low (Connely, 2015), and therefore, nursing was often viewed as a unique work opportunity for independent and adventurous women (Rutherdale, 2010).

At the same time, governments expected nurses to function in an expanded role that could fulfill most healthcare needs of the population at a much lower cost than physician services (Gilbert, 2003; Vandenberg & Johnson, 2022). Many mid-century outpost nurses were young, female, European-Canadian, English-speaking, middle-class, and university-educated with little preparation for the cultural diversity they experienced in practice (Drees & McBain, 2001; Elliott, 2008). Administrators expectations for ‘low-cost’ nurses to ‘fill-the-gap’ in healthcare in these remote locales were common (Vandenberg & Johnson, 2022).

Like nurses during the postwar period, rural and remote of the twenty-first century continue to face increasingly complex patients transferred from early discharge programs and off-sited services used to optimize urban bed capacity (MacLeod et al., 1998). Rural and remote nursing continues to require a generalist skill set accompanied by expanded knowledge requirements and productivity demands (MacLeod et al., 1998, 2019a). Rural nurses report working to a full or extended scope without additional educational or practice support (MacLeod et al., 2019a, 2019b; Martin-Misener et al., 2020). Intent to leave is associated with rural nurses working outside of their scope of practice, not feeling prepared for their scope of practice, and having low satisfaction with their practice and the support available to them (Stewart et al., 2020). To some extent, it has been argued that technology now provides staff with access to practice support resources and efficient telehealth communication systems that decrease professional isolation (MacLeod et al., 2019a, 2019b; Stewart et al., 2020).

At present, MacLeod et al. (2017) noted that most RNs in rural and remote regions are female, diploma-trained, and married or living with a partner. Fewer than 7% of rural nurses have Indigenous ancestry, which is slightly higher than the national percentage (MacLeod et al., 2017). Yet influenced by current trends in critical and post-colonial theory, clear expectations for enhanced understanding of and cultural sensitivity towards diverse populations is a key priority of contemporary nursing education. Rural nurses are uniquely challenged in their ability to maintain patient confidentiality and nursing professionalism in rural settings where community members are often closely acquainted (MacLeod et al., 2019a).

### The complexity of rural and remote recruitment and retention

The complexity of rural and remote nursing and the diversity of geographical, population, and organizational practice contexts suggest that there is no “one-size-fits-all” solution to recruitment and retention challenges (Stewart et al., 2020). Historical (n = 4) and contemporary (n = 5) scholars have identified rural and remote nursing as a complex subspecialty of nursing that requires a generalist knowledge and skill set (Drees & McBain, 2001; Elliot, 2010; Kulig et al., 2015; MacLeod et al., 1998, 2019a; Martin-Misener et al., 2020; Quiney, 2008; Stewart et al., 2020; Vandenberg & Johnson, 2022). Researchers have difficulty studying the migration of rural and remote nurses due to the lack of congruent definitions (Martin-Misener et al., 2020) and subsequent sparsity of accurate data to reflect their migration patterns (Pitblado et al., 2005). Several demands are unique to rural and remote nursing and differentiate it from other subspecialties and areas of nursing practice.

#### Unique demands of rural and remote nursing

The unique demands of rural and remote nursing affect the transferability of policy, practices, and education systems influenced primarily by those in urban settings. Each rural community plays an integral part in the recruitment and retention of healthcare professionals (Kulig et al., 2009; MacLeod et al., 2019a; Rohatinsky et al., 2020; Stewart et al., 2011, 2020). Geography separates rural communities from the social constructs, infrastructure, and amenities associated with urban centers. Without connections to the community, personal relationships, and professional relationships, rural nurses experience social isolation, job dissatisfaction, loneliness, and intent to leave (Kulig et al., 2009; MacLeod et al., 1998, 2019a; Rutherdale, 2010). The interconnection between community engagement, workplace engagement, and self-care nurture professional confidence and competence (MacLeod et al., 2019a). The need to work long hours (Quiney, 2008) and assume on-call responsibilities impact the work-life balance and job satisfaction of rural nurses (MacLeod et al., 2017; McBain, 2012; Stewart et al., 2011). Rural nurses also spend considerable time travelling to geographically dispersed populations (Martin-Misener et al., 2020; Quiney, 2008). Modern vehicles have improved transportation and access to rural and remote communities; however, travel can still be challenging. Transportation may be restricted by weather, geography, lack of infrastructure, or efforts to contain the costs of extending healthcare to remote communities (McBain, 2012).

A good rapport with local populations is as important as professional competency in rural communities (Elliot, 2010). Building a therapeutic relationship with populations can be challenging if the actions and behaviors of the nurse do not coordinate with those of the locals (Quiney, 2008). Nursing historians have often observed that nurses who approached Indigenous communities with openness, curiosity (Elliot, 2010, Rutherdale, 2008; Rutherdale, 2010), and cultural sensitivity were more successful in building relationships based on mutual learning and collaboration (Drees, 2010).

Historically, rural and remote nurses experienced poor living and working conditions, especially nurses in more northern regions (McBain, 2010, 2012; Quiney, 2008). Rural healthcare facilities often lacked sufficient health human resources (Ariste, 2019), practice support resources (MacLeod et al., 1998; Stewart et al., 2005), and overall health and social support systems available in urban areas (Martin-Misener et al., 2020). Nurses often assumed the role of “gap-filling” (Martin-Misener et al., 2020) within the healthcare system to compensate for resources or services that would otherwise be unavailable to the patient in a rural context (Vandenberg & Johnson, 2022). Tasks that are completed by support staff or other professionals are frequently shifted onto nursing, contributing to an increased workload, and an expanded spectrum of skills required to practice competently (Martin-Misener et al., 2020). Due to the ongoing resource limitations, gap-filling and task shifting are phenomena that continue to impact rural nursing practice (Martin-Misener et al., 2020; Montour et al., 2009).

### Strategies to support recruitment and retention of rural and remote nurses

Kulig et al. (2015) suggest that most recruitment and retention strategies utilize a combination of educational opportunities, financial incentives, and enhanced infrastructure for workplaces and families. Yet the literature is limited in that rural recruitment and retention initiatives suffer from a lack of evaluation and long-term implementation feedback (Kulig et al., 2015). This limitation is evidenced by only one of the 27 pieces of literature including data on the implementation and evaluation of a specific strategy to support the recruitment and retention of rural and remote nurses (Rohatinsky et al., 2020). The unique demands of rural and remote practice settings offer extremely limited capabilities for data collection and project management unless additional support is to be dedicated to that function (Montour et al., 2009; Pitblado et al., 2005).

#### Educational opportunities

Of the 27 articles included, 22 directly or indirectly discussed the role of nursing education systems in sustaining Canada's rural and remote workforce (Ariste, 2019; Drees, 2010; Drees & McBain, 2001; Elliot, 2010; Kulig et al., 2015; MacLeod et al., 1998, 2017, 2019a, Martin-Misener et al., 2020; McBain, 2010, 2012; Pitblado et al., 2005; Quiney, 2008; Richardson, 2001; Rohatinsky et al., 2020; Rutherdale, 2010; Stewart et al., 2005, 2011, 2020; Tallman & Bruning, 2005; Tipliski, 2004; Vandenberg & Johnson, 2022). One of the most common reasons a nurse leaves a rural setting is to pursue higher education (Pitblado et al., 2005; Stewart et al., 2011). Many nurses who leave rural and remote settings to pursue higher education never return (Stewart et al., 2005). Scholars suggest that rural nursing education programs, Indigenous-focused education programs (Drees, 2010; MacLeod et al., 1998), mentorship programs (Rohatinsky et al., 2020), and distance education programs (Stewart et al., 2011) support rural recruitment and retention by allowing future students and current professionals to learn in place without relocation (Ariste, 2019; Kulig et al., 2015; MacLeod et al., 2019a).

#### Financial incentives

Financial incentives are a common recruitment strategy used to attract employees to areas deemed less desirable. Thirteen articles referred to either the use of or lack of financial incentives in supporting rural nursing recruitment (Ariste, 2019; Drees, 2010; Elliott, 2008; Gilbert, 2003; Kulig et al., 2015; MacLeod et al., 2017; McBain, 2012; Quiney, 2008; Richardson, 2001; Rutherdale, 2010; Stewart et al., 2005; Tallman & Bruning, 2005; Vandenberg & Johnson, 2022). Historically, rural nurses have received poor financial compensation in comparison to male-dominated professions like medicine (Elliott, 2008; McBain, 2012; Quiney, 2008; Richardson, 2001). Rural nursing often attracted single women or those from impoverished backgrounds because it offered subsidized education, free room, and board during schooling, subsidized housing at outpost locations, a livable wage, and access to domestic help and transportation that would otherwise be unaffordable (Elliot, 2010, Elliott, 2008; Rutherdale, 2010). Contemporary scholars report fewer financial incentives of this nature, but more commonly individual provinces and territories use loan forgiveness and grant programs to support recruitment and retention (Kulig et al., 2015).

#### Enhanced infrastructure

Initiatives to support recruitment and retention through enhanced infrastructure include improvements to rural healthcare delivery, public policy, education systems, community amenities, and community engagement with healthcare providers (Kulig et al., 2015). All 27 pieces of literature included observations surrounding the systemic inadequacy of infrastructure to support rural healthcare or recommendations regarding what enhancements will best support rural nursing recruitment and retention. Multiple scholars have reported the significant role that organizational factors have in influencing rural nurses’ job satisfaction and intent to leave their current workplace or the profession (Elliot, 2010; McBain, 2012; Montour et al., 2009; Stewart et al., 2020; Tallman & Bruning, 2005). Practice supports such as mentorship (Rohatinsky et al., 2020), supportive managers (Tallman & Bruning, 2005), and lower on-call commitments are suggested along with measures to increase community engagement (Kulig et al., 2015; Stewart et al., 2020).

### Rural marginalization on rural nursing recruitment and retention

The contextual background information provided in this review alluded to the health and socioeconomic disparities experienced across rural areas of Western Canada. Ten papers conducting historical inquiry discussed how policymakers overlooked the needs of rural communities and how rural regions often received inequitable proportions of government funding and physical resources, such as medications (Drees, 2010; Drees & McBain, 2001; Elliot, 2010, Elliott, 2008; Gilbert, 2003; McBain, 2010, 2012; Quiney, 2008; Rutherdale, 2008; Vandenberg & Johnson, 2022). The sparsely distributed populations, diffuse poverty, poor access to education, and precarious work meant that most rural communities could not afford to sustain a nursing outpost or fund improvements to existing infrastructure (McBain, 2010, 2012; Quiney, 2008). Chronic underfunding and indifference to rural needs have resulted in the continued marginalization of these areas (Drees, 2010; Drees & McBain, 2001; Elliot, 2010, Elliott, 2008; Gilbert, 2003; McBain, 2010, 2012; Quiney, 2008; Rutherdale, 2008). Seven recent articles noted the lack of attention, data, and resources for rural healthcare practice, policy, and research (Kulig et al., 2015; MacLeod et al., 1998, 2017; Martin-Misener et al., 2020; Pitblado et al., 2005; Stewart et al., 2011, 2020). Martin-Misener et al. (2020) importantly note that without consistent definitions and data to describe rural and remote nursing practice, rural communities lack the concrete evidence to advocate for additional funding and resources.

Nine historical articles noted that the racial and geographical marginalization, which stemmed from colonialist attitudes and social systems that were dominant throughout the twentieth century, created challenging local conditions and broader political barriers for nurses to navigate while trying to improve the health of rural communities (Drees, 2010; Drees & McBain, 2001; Elliott, 2008; Gilbert, 2003; McBain, 2010, 2012; Quiney, 2008; Rutherdale, 2008, 2010). Colonialism in the nineteenth and early twentieth century involved groups of British-Anglo settlers exerting political control over Indigenous and various immigrant groups to support forced assimilation or “Canadianizing” (Quiney, 2008, p. 93; Vandenberg & Gallagher-Cohoon, 2021). Conflicts regarding immigration labour, forced displacement of Indigenous groups, and provision of funding for Indigenous healthcare in Canada are complex problems influencing rural Canada (Drees, 2010; Gilbert, 2003) that extend beyond the scope of this review.

### Political influences on rural nursing recruitment and retention

One prominent theme from the historical literature was the influence that government had on the operations, and therefore the nursing workforce, of rural and remote outposts and healthcare facilities. Governments utilized nurses as agents of change in rural and remote Canada (Drees & McBain, 2001; McBain, 2010; Quiney, 2008; Rutherdale, 2008). In this role, many nurses were positioned as both conveyors and recipients of colonial attitudes directed towards the citizens they cared for (McBain, 2010). In the 1920s-1930s, political agendas to spread “civilization” and improve rural economies lead to the development of the outpost nursing model (Drees & McBain, 2001; McBain, 2012; Quiney, 2008; Rutherdale, 2008). Regional supervisors enforced high expectations and meager resources on nurses working for the Canadian Red Cross Society (CRCS). They censored complaints or requests by nurses that could impact budgets or threaten the reputation of the organization, including requests to transport ill patients to higher levels of care (McBain, 2012). Nurses were subject to frequent transfers and denied the right to resignation to control their behavior while retaining their labor (McBain, 2012). Many nurses emigrated to the United States for better salaries and working conditions, while others left the profession for marriage or other careers (Richardson, 2001).

During the mid-twentieth century, Canada's Indian Health Service Branch often hired health professionals in cities far away from the communities they served (Drees, 2010). As a result, hiring tendencies favored foreign nationals and non-Indigenous workers rather than supporting the development of Indigenous professionals and culturally integrated healthcare (Drees, 2010). Contemporary literature, other than that focusing on Indigenous health history, does not frequently discuss the impact of these legacies on healthcare in Canada (Gilbert, 2003).

Governments have repeatedly pushed nursing associations to reduce the educational requirements for healthcare providers as a solution to recruitment and retention challenges. Following WWII and up until the 1960s, the conservative Social Credit party in Alberta restricted the separation of nursing schools from primary hospitals (Richardson, 2001). Amidst post-war nursing shortages, political leaders were more concerned with preserving the supply of student labor than ensuring that the minimum standards of nursing education were being met (Richardson, 2001). In the early 2000s, several Western Canadian governments promoted shorter diploma programs as a practical solution to nursing shortages to produce nurses more cheaply, much to the dismay of Canada's Nursing Associations (Tipliski, 2004). Nurses in Canada continue to be challenged today to provide quality care within cost-containment organizational cultures (Kelly & Porr, 2018).

### Urban majority mindset and subsequent power imbalances in rural settings

Throughout history, social structures centered around gender, region, race, class, and professional power hierarchies have strongly influenced the nursing profession (Drees, 2010; Elliot, 2010, Elliott, 2008; Gilbert, 2003; McBain, 2012; Quiney, 2008; Richardson, 2001; Rutherdale, 2010; Tipliski, 2004; Vandenberg & Johnson, 2022). In the early twentieth century, nursing was primarily aimed at white middle-class and working-class women (Gilbert, 2003; Drees & McBain, 2001; Quiney, 2008; Tipliski, 2004). In some instances, the power imbalances between physicians and nurses could be amplified in rural settings where rural physicians often faced no consequences for poor behavior, while organizations held nurses to high moral standards and used them as scapegoats for compromised patient care (Elliot, 2010).

Rural healthcare is shaped by an organizational design of Canadian healthcare that favors the urban majority. The intrinsic connection of politics and healthcare gives democratic policy-making power to densely populated urban areas. As a result, organizational designs, public policy, and education systems often fail to meet the needs of diverse rural and remote populations (Drees & McBain, 2001; McBain, 2010, 2012; Montour et al., 2009; Pitblado et al., 2005; Vandenberg & Johnson, 2022). For example, during the interwar period in British Columbia, healthcare leaders promoted hospital standardization to bring rural hospitals ‘up to’ urban standards (Vandenberg & Johnson, 2022). The idealization of urban hospital standards highlighted the power imbalances that existed between medical leaders, the nursing elite, and the rank-and-file nurses operating rural hospitals on the periphery (Vandenberg & Johnson, 2022). Male physicians dominated the discussion at the British Columbia Hospital Association conventions, where frontline nurses’ requests for increased education and resources to support their expanded scope of practice in rural settings were dismissed in favor of nursing leaders’ goals of professional advancement and medical leaders’ goals of standardization (Vandenberg & Johnson, 2022). These priorities shifted more responsibilities, such as record keeping and diagnostic services, onto rural nurses without any additional recognition, compensation, or education for the additional workload expectations (Vandenberg & Johnson, 2022). By favoring urban needs, organizational structures increased the barriers for nurses trying to meet the specific needs of diverse rural communities. These historical legacies still influence and sustain the lack of contextually-based care in rural areas.

Three historical articles identified the nursing profession's struggles with authority over nursing education and practice domains (Tipliski, 2004; Richardson, 2001; Vandenberg & Johnson, 2022). In the past, professional associations often positioned elite nursing leaders to advocate on behalf of the rank-and-file professional body, but the goals of these groups often differed (Vandenberg & Johnson, 2022), and at present many professional bodies no longer serve an advocacy function. The public needs to hear the voices of rural and remote nurses, but they also must be more cognisant of the power they yield over rural and remote patient populations (Rutherdale, 2008, 2010). The high degree of professional autonomy associated with rural practice comes with great responsibility.

## Discussion

The complexity of rural and remote nursing suggests that there is no “one size fits all” solution to nursing recruitment and retention challenges (Stewart et al., 2020). The synthesis of the findings in this review reveals how a lack of attention to historically and socially constructed contextual inequities intersect to sustain recruitment and retention as a significant problem in Western Canada.

### Communicating the rural context

Experts in rural nursing scholarship suggest that future initiatives should focus on the strengths of rural communities and rural nursing practice (Kulig et al., 2015; Stewart et al., 2020). History has shown us that recruiters must market the strengths of rural and remote communities with realistic and honest information. Twentieth-century outpost nurses often developed feelings of self-doubt and job dissatisfaction due to the incongruencies between what was taught and what was experienced in remote communities (Elliot, 2010; McBain, 2010). Recruitment efforts that glorify northern nursing and capitalize on the exotic beauty of Indigenous/ Immigrant people and lifestyles may unintentionally endorse the colonial systems and attitudes that threaten these populations’ health (Gilbert, 2003). Nursing education and recruitment marketing that portrays rural nursing in a realistic and culturally sensitive way offers nurses an unambiguous foundation for future rural practice.

### The unique professional, organizational, and educational needs of rural nurses

Contemporary scholars continue to identify rural and remote nursing as a unique generalist subspecialty of practice that requires nurses to confidently function to a full or extended scope of practice in a variety of settings. However, there is still a need to improve educational supports that do not strip educational interventions of contextual differences. Current systems ought to consider the drawbacks of promoting a ‘gold standard’ of urban ideals and ‘one-size-fits-all’ approach for nurses who will potentially work in rural contexts.

This review reveals that registered nurses raised in rural areas tend to stay in rural practice settings and thus educational institutions ought to target post-secondary education to rural and remote students (Ariste, 2019; Drees, 2010; Kulig et al., 2009). A recent systematic review of rural and remote recruitment among physicians suggests that the most effective interventions begin as earlier interventions before graduation, despite the current widespread use of financial incentives by many provincial governments (Lafortune & Gustafson, 2019).

### Social, political, economic, organizational, and professional marginalization of rural and remote nursing

Rural nurses have long been subjected to maltreatment from healthcare organizations and governments compared to urban nurses because of their association with “charity” and the lack of solidarity needed to advocate for reform. Cost containment continues to be the major priority of current neoliberal governments, resulting in funding restrictions that disproportionately impact rural regions and perpetuate marginalization. Formally engaging communities in recruitment and retention issues can help to sway political powers to take note of rural healthcare issues. “Band-aid” policy solutions, such as international nursing recruitment, to compensate for short-term labor deficiencies ought to be cautioned to refocus on the root causes of this issue.

Lastly, the lack of financial support for research, data collection, and subsequent evaluation of rural and remote nursing practice continues to contribute to this problem. More longitudinal formal evaluations of recruitment and retention initiatives are needed to better understand future directions for policy, education, and research. Leatt et al. (2000) suggest working towards integration by using a patient focus, starting with primary care, sharing information, exploiting technology, creating virtual networks at the local level, implementing needs-based funding structures, and designing mechanisms to monitor and evaluate system progress to support more sustainable rural health systems.

## Conclusion

An exploration of the persistent challenges of rural and remote nursing recruitment and retention in Western Canada reveals a lack of thoughtful attention to the past ideologies sustaining healthcare staffing shortages in rural Canada. Addressing rural marginalization, cost-containment organizational cultures, lack of development of the nursing profession, urban majority mindset, and poor organization design may be integral in developing effective and sustainable solutions to support rural and remote nursing recruitment and retention. Communities, healthcare organizations, and educational institutions must join forces to challenge the pervasive ideologies that ignore the complexity and diversity of rural health needs. Short-term solutions, including financial incentives, will likely fail to address the long-standing challenges that perpetuate nursing shortages in rural and remote areas of Western Canada.

## Supplemental Material

sj-docx-1-cjn-10.1177_08445621231204962 - Supplemental material for Help Wanted, Experience Preferred, Stamina a Must: A Narrative Review of the Contextual Factors Influencing Nursing Recruitment and Retention in Rural and Remote Western Canada from the Early Twentieth Century to 2023 Supplemental material, sj-docx-1-cjn-10.1177_08445621231204962 for Help Wanted, Experience Preferred, Stamina a Must: A Narrative Review of the Contextual Factors Influencing Nursing Recruitment and Retention in Rural and Remote Western Canada from the Early Twentieth Century to 2023 by Amanda M. McCallum, Helen E. R. Vandenberg and Kelly L. Penz in Canadian Journal of Nursing Research

## Appendix A

**Table 1.** Summary of studies included in review.

Source	Design or Methodology, & Data Collection	Objective/ Purpose	Sample/ Target Population	Key Findings	Recommendations
Contemporary content					
MacLeod, M., Browne, A. J., & Leipert, B. (1998). Issues for nurses in rural and remote Canada.	Scholarly Article	Describe the context of rural and remote nursing practice in Canada and issues of health status, social determinants of health, geographical/ professional isolation, and cultural safety.	Rural and remote Canada, Indigenous populations	Changing organization of healthcare. Nurses are primary healthcare providers with broad responsibilities and serve large areas/ several communities. Issues include: tailor practice to needs of community, burden of responsibility, generalist or multi-specialist roles, knowledge demands, lack of resources, personal and professional isolation. Rural nursing is not generalist nursing, it is a complex specialty area.	Importance of cultural safety in Indigenous communities. Nurse managed care initiatives and/or utilization of NP's. Programs to recruit nursing students (and Indigenous students) from rural and remote areas. Programs designed for rural nurses. More effort needed to develop effective rural healthcare policy and research.
Rohatinsky N, Cave J, Krauter C. (2020). Establishing a mentorship program in rural workplaces: connection, communication, and support required.	Research Article, Qualitative, Post- Intervention Survey and Interview	Describe and evaluate a rural mentorship program in terms of supporting rural mentorship, easing workplace transition, strengthening community connections, and encouraging recruitment and retention in rural communities.	Purposeful Sampling, N = 30 Rural (<10,000), Western Canada RNs	Three themes related to mentorship: Connection: to mentor/mentee, themselves, profession, colleagues, and larger rural community. Communication: between mentor/ mentee, with coordinators of program, about program, and promotion of future programs. Support: interpersonal and interprofessional assistance from mentor, mentorship program, and management.	Mentorship is valuable Need commitment from the mentors, mentees, the healthcare organization, and rural communities for sustainability.
Stewart, N. J., D’Arcy, C., Kosteniuk, J., Andrews, M. E., Morgan, D., Forbes, D., MacLeod, M. L., Kulig, J. C., & Pitblado, J. R. (2011). Moving on? Predictors of intent to leave among rural and remote RNs in Canada.	Research Article, Quantitative, Cross-Sectional Survey	Explore predictors of intent to leave a nursing position in rural and remote practice settings in Canada.	Stratified random sampling, Subsample (n = 3,051) RNs in rural and remote Canada, Logistic Regression Analysis	Significant predictors of intent to leave include male gender, perceived stress, no dependent children or relatives, highest attained nursing education, fewer years employed, lower community satisfaction, scheduling dissatisfaction, work satisfaction autonomy, required to be on call, performed advanced decisions or practice, and workplace remote.	Determinants related to individual, the workplace, the community, and satisfaction levels to guide policy makers and employers in developing retention strategies. Sustainability of rural nursing workforce must address nurses’ perceptions of the environment at the community and workplace level. Importance of distance education options for rural and remote RNs.
Kulig, J. C., Stewart, N., Penz, K., Forbes, D., Morgan, D., Emerson, P. (2009). Work setting, community attachment, and satisfaction among rural and remote nurses.	Research Article, Quantitative, Cross-Sectional Survey	To describe community satisfaction and attachment among rural and remote registered nurses in Canada.	Stratified random sampling, Subsample (n = 3,331) Rural and remote RN's in Western Canada, Northern regional areas, and outposts.	Greater community satisfaction associated with home care and community health RN's, being raised rurally, and larger rural communities. Community attachment: going home and becoming home. Community satisfaction: being rural and becoming rural.	Recruitment and retention strategies need to include mechanisms that focus on community satisfaction and attachment. Increased community involvement to support attachment of new nurses and increased efforts to recruit local high school students into nursing.
Stewart, N. J., MacLeod, M. L. P., Kosteniuk, J. G., Olynick, J., Penz, K. L., Karunanayake, C. P., Kulig, J. C., Labrecque, M. E., Morgan, D. G. (2020). The importance of organizational commitment in rural nurses’ intent to leave.	Research Article, Quantitative, Cross-Sectional Survey	To examine determinants of intent to leave (ITL) rural and remote nursing positions within the next year.	Stratified random sampling, Subsample (n = 1,932 RN's/ NPs, n = 1,133 LPNs) RNs and LPNs, Rural and Remote Canada	Organizational commitment was the only variable associated with ITL for RNs and LPNs. Factors associated with increased ITL for RN's include being over age 60, no dependent children, experiencing higher perceived stress, working beyond or below scope of practice, low job satisfaction, low organizational commitment, low preparedness for their scope of practice, long commutes to work, on-call requirements, lack of work flexibility, and lower community satisfaction.	Suggestions include specific continuing education initiatives, mentoring programs, and career pathways. Place based actions to enhance nurse's integration into the community. Work systems need to suit realities of local contexts to work well and be supportive. Practice supports, supportive managers.
Tallman, R., Bruning, N. S. (2005). Hospital nurses’ intention to remain: Exploring a northern context.	Research Article, Quantitative, Cross-Sectional Survey	Identifying factors that relate to nurses’ intentions to remain in northern and rural hospitals which managers can influence.	Convenience sampling, N = 122, Northern Western Canada Hospital Nurses, Management	Affective commitment, continuance commitment, and ties to the community significantly related to intentions to remain. Ties to the community was strongest predator of intent to remain. Four components with strongest relationship to job satisfaction include: positive anticipation of going to work, a safe environment, utilization of knowledge and abilities, and how nurses were treated by management.	Nurturing local talent with ties to the community may increase retention. Commitment to stay is heavily influenced by the way managers treat staff and the organizational climate they create. Most important factors for managers to consider are safety of the work environment, their actual and perceived view of nurses, and fairness of policies.
Stewart, N. J., D'Arcy, C., Pitblado, J. R., Morgan, D. G., Forbes, D., Remus, G., Smith, B., Andrews, M. E., Kosteniuk, J., Kulig, J. C., & MacLeod, M. L. (2005). A profile of registered nurses in rural and remote Canada	Research Article, Quantitative, Descriptive Questionnaire	Determine who practices nursing in rural and remote Canada, the nature and scope of their practice, satisfaction with their work, community, and practice supports.	Stratified random sampling, N = 3,933 Rural and Remote RNs Canada	In western provinces, lower percentage of young nurses and higher percentage of retirement age nurses were working in rural and remote areas. Higher percentage of western nurses working in LTC and homecare and lower percentage in hospitals. RNs in AB and BC reported higher mean work satisfaction. Nurses in AB had higher satisfaction with pay. Western provinces had moderate community satisfaction.	Improvements in education and interdisciplinary support for practice to target needs of rural and remote nurses. Organizations need increased health human resource planning. Higher pay was a contributor to work satisfaction in this study and may be an important factor to consider, especially for young new graduates.
Pitblado, J. R., Medves, J. M., & Stewart, N. J. (2005). For work and for school: Internal migration of Canada's rural nurses	Research Article, Multi Method, Database and Cross-Sectional Survey	Explore the internal migration patterns of Canadian-educated, rural RNs.	Stratified random sample, Subsample (n = 3,460) Rural RNs, Canadian educated, Canada	Migration patterns of rural RNs include movement to adjacent provinces, movement to larger magnet provinces of ON, AB, and BC, and an east to west flow. Rural RNs most likely to migrate are female, older age, working in nursing stations, and living in more remote communities. Many nurses move interprovincially to attain higher education after initial nursing education and many don’t return.	Lack of operational data on how presumed factors (i.e.,: work FTE) influence migration trends. There is a need for migrant specific studies and data, as currently no way to track nursing migration interprovincially. Workforce planning regarding migration necessary to protect vulnerable communities from shortages due to increasing numbers of retirement.
Kulig, J. C., Kilpatrick, K., Moffitt, P., & Zimmer, L. (2015). Recruitment and retention in rural nursing: It's still an issue!.	Scholarly Article, Documentary Analysis	Discuss initiatives identified in a documentary analysis review following Rist's (1994, as cited in Kulig et al., 2015) components of the policy cycle regarding the ongoing issue of recruitment and retention of rural and remote nurses in Canada.	Rural and Remote, Canada, RNs	Proposed solutions fall into 3 categories: educational opportunities, financial incentives, and enhanced infrastructure for the workplace and to support families. No formal evaluations of any recruitment or retention strategy. Policy formulation, implementation and accountability are important but are not often formally assessed when reviewing reports.	Collaboration between communities and government to ensure successful recruitment and retention to rural and remote communities. Viewing rural healthcare from a strengths-based framework. Formal evaluation is needed to guide implementation and increase accountability. Collaborations between various sectors to develop more viable and sustainable solutions.
MacLeod et al. (2017). Nurses who work in rural and remote communities in Canada: A national survey.	Research Article, Quantitative, Cross-sectional survey	Describe the study and examine the characteristics of the rural and remote nursing workforce with a focus on important variations among nurse types and regions.	Stratified systematic sampling, N = 3,822 Rural and remote Canada, <10,000, RNs, RPNs, NPs	Rural workforce characteristics include aging rural nursing workforce, growth in baccalaureate education for RNs, and increasing casualization. Top 3 recruitment and retention factors were: location of community, interest in the practice setting, and income. Intent to leave factors were lack of work flexibility, stress, and requirement to be on call.	Further analysis about workplace demands, legislation, organizational, and community challenges across Canada to identify potential approaches to enhance rural recruitment and retention. Discourses on the strengths of rural communities to maximize potential to increase and stabilize workforce.
MacLeod, M., Kulig, J., & Stewart, N. (2019). Lessons from 20 years of research on nursing practice in rural and remote Canada.	Scholarly Article, Personal Reflections of Research	Describe lessons from 20 years of research on rural and remote nursing practice in Canada.	Rural and Remote Canada, RNs	Challenges in providing appropriate education, educating nurses in northern communities, and having sufficient amenities in very small communities to attract and retain nurses and their families. Rural nurses remain generalist, must be flexible, innovative, leaders, and support the health of the community. Confidence and competence depended on interconnection of nurses’ engagement in community, engagement in the workplace, and keeping well. Further enhanced through interprofessional collaboration, teamwork, a professional support network, involvement in leadership activities.	Recruiting nurses from smaller communities and offering welcoming strategies to all newly arrived nurses to help transition and community engagement. Few policies and guidelines are tailored to the realities of rural and remote contexts. Leaders and health organizations must address resource limitations. Rural and remote nurses need to be recognized and appreciated by employers and the communities. Ongoing identification of characteristics of rural and remote practice, opportunities, and challenges in research and policy.
Martin-Misener, R., Macleod, M. L. P., Wilson, E. C., Kosteniuk, J. G., Penz, K. L., Stewart, N. J., Olynick, J., Karunanayake, C. P. (2020). The mosaic of primary care nursing in rural and remote Canada: Results from a national survey	Research Article, Quantitative, Cross-Sectional Survey	Examine the practice context and responsibilities of primary care nurses in rural and remote Canada.	Stratified systematic sampling, N = 3,822 Rural and Remote Canada, NPs, RNs, LPNs, Primary Care (PC)	Approximately half of rural PC nurses worked in multiple areas. Working PC only was associated with larger rural communities closer to urban centers. PC-Plus nurses reported better fit between workplace and community needs but had higher job-related demands and fewer job-related resources. No significant difference between groups regarding comfort, competence, and confidence. Task shifting and gap filling phenomena	Education and regulation to cover RNs whose work involves filling gaps in absence of other professionals. More NPs and increased practice supports to reduce the double standard of care between urban and rural. Expanding CIHI definition of PC to be more inclusive of rural and remote workplaces to allow for data consistency and future research and policy advancement.
Ariste, R. (2019). Availability of health workforce in urban and rural areas in relation to Canadian seniors.	Research Article, Quantitative, Database/ Statistical Areas Classification Analysis	Determine extent to which the distribution of regulated health professionals and seniors in urban and rural areas of the Canadian jurisdictions is different from one another and the national average.	Canada, urban versus rural, health professionals, seniors	Twice as many nurses/1000 seniors in urban Canada than rural Canada, 3 times as many physicians/ 1000 seniors in urban versus rural Canada. BC and SK have low availability of nurses in rural areas, MB has good availability and distribution, AB around national average but lower in suburban areas. Rural areas in BC are better served with GPs than MB, SK, or AB suburban areas.	Three strategies include: Target current nurses and physicians (financial and logistic incentives). Target future nurses and physicians (education programs, create positions with balanced hours and compensation, consider non-academic admission criteria). To do with less (rural health system redesign, maximize scope, utilize alternate healthcare professionals/ support staff, utilize technology, innovative solutions).

Source	Type	Objective/ Purpose	Sample/ Target Population	Key Findings or Themes	Recommendations
Historical Content					
Vandenberg, H., Johnson, L. (2022). Filling the gap between metropoles and peripheries: Insights about hospital standardization from the British Columbia Hospital Association conferences, 1918–30	Scholarly Historical Original Research Paper	To understand how place, gender, and profession shaped debates about hospital standardization during the interwar period in British Columbia.	British Columbia, Nurses 1920s-1930s Interwar Period	Hospital affiliation used to bring rural nurses to urban hospitals for training. (urban superiority). Aligned with the goals of nursing elite for standardization and ignored the actual needs of small community hospitals. Complexity of budgeting variance, expenditures, and resource allocation between urban and rural and geographically remote. Often suggested that rural nurses could fill gaps such as administrative duties, to reduce costs. Gendered, spatial, and professional biases or hierarchies within BCHA.	Professional goal of advancing nursing education in Canada overshadowed the voices of rural rank and file nurses Increased demands on rural nurses was not accompanied by increased funding or salary. Generalist demands of rural nurses. Rural nurses wanted more professional education to support doing the same.
Richardson, S. L. (2001). “Stand up and be counted”: Nursing at the Calgary General Hospital of the Second World War	Scholarly Historical Original Research Paper	A case study of how the Calgary General Hospital-grappled with the post-second World War shortage of graduate and student nurses.	Calgary General Hospital, Western Canada	Government restricted the separation of nursing education from hospitals until the 1960's. Government valued student nurse supply for staffing more than meeting minimum nursing education standards. Prior to public health insurance, challenge to fund consistent and sustainable healthcare in any hospital or community. Nursing crisis at CGH largely due to poor organization and operations, not just the postwar nursing shortage. Nurses used threats of “strike” to prompt the hospital board to change.	Staffing based on job analysis of nurses’ work; auxiliary personnel for domestic and repetitive tasks; clinical rotations of student nurses planned to meet their learning needs, rather than the service needs of the hospital; and adequate financing of hospitals to enable them to care for the sick. Reform of CGH included separation of nursing training from hospital, hospital wide surveys to assess need for change, effective communication, organization and appropriate delegation, professional development incentives, length of service salaries, fair shift rotations, and professional development. Collaboration between hospital and school of nursing.
McBain, L. (2012). Pulling up their sleeves and getting on with it: providing health care in a northern remote region	Scholarly Historical Original Research Paper	Illustrate the complexities of providing nursing care in Northern Saskatchewan outpost nursing stations between mid 1940s and 1950s.	Northern Saskatchewan, Remote, Outpost Nurses.	Nurses had a complete lack of power. Complaints and requests often downplayed or dismissed by supervisors to minimize budgets. Difficult to recruit and retain nurses to work in the north during times of general nursing shortages, resignations, and poor working conditions. Nurses received no support for language or cultural orientation. Nurses were frequently transferred, challenging to build relationships. Rural nurses expected to live in poverty with no regard for personal welfare, substandard pay, and resignations were not allowed.	Nurses often forced to work beyond scope because of no physician availability. Government allocated disproportionately low educational, health, water, and sanitation infrastructure to northern communities. Restricting potential for improvement of the social determinants of health and increasing marginalization. Recollections of outpost nurses often glorify the trials they faced, but we should be looking at why such conditions were allowed to exist.
Tipliski, V. M. (2004). Parting at the crossroads: The emergence of education for psychiatric nursing in three Canadian provinces, 1909–1955.	Scholarly Historical Original Research Paper	How did Canadian psychiatric nursing develop into two completely different models that switch at the Manitoba/ Ontario border during the?	RNs, RPNs, Western Canada, twentieth century	Interplay of social, economic, and political factors influenced development of psychiatric nursing. The psychiatrist-controlled apprenticeship programs of Western Canada stopped at the Manitoba/ Ontario border. Suggests organized nursing continues to struggle with issues of autonomy and authority over education and practice.	Suggests that the attempt from psychiatrists to spread the Western Canadian Model of training nationally, to solve the country's mental hospital nursing shortage, parallels the modern struggles between government and nursing associations over the models of education to best address current (2004) nursing shortages (i.e.,: diploma or degree).
Gilbert, H. (2003). Great adventures in nursing: Colonial discourse and health care delivery in Canada's north	Scholarly Historical Original Research Paper	Discuss colonization and health care delivery in Canada's North.	Canada's North, Outpost Nursing, Indigenous populations	The outpost model was adopted for the north because of economics and gender and class perceptions. Government allows communities minimal input into health policy and administration. Recruitment efforts have glorified northern nursing, which may contribute to later dissatisfaction, high turnover, and perpetuates colonialist attitudes and practices.	Idealization and misrepresentation of culture, populations, and nursing profession. Need to consider the harm that the oppressive medical hierarchy can cause in the context of Indigenous healthcare when care is not given to integrate the services to best meet the holistic needs of the patients.
Drees, L. M., & McBain, L. (2001). Nursing and Native Peoples in Northern Saskatchewan: 1930s–1950s	Scholarly Historical Original Research Paper	Investigate the role of nurses in northern Saskatchewan Aboriginal communities in Canada between 1930 and the 1950s.	Canada, Northern Saskatchewan, Outposts, Nurses	Predominantly Euro-Canadian, English, university educated, middle class, single, served 2–3 year terms. Outpost nurses practiced in expanded roles, received no cross-cultural or language training, did not learn local norms of health and wellness, did not integrate with communities, and were socially isolated. 1950s- federal and provincial grants for nurses to increase training. Nursing shortages limited expansion of nursing outposts. Nurse in role of authority, was not questioned by Indigenous people	Lack of research on the role of nurses in the delivery of Indian healthcare services. Effort to integrate the medical model and traditional health practices. The weak commitment of Federal and Provincial governments to providing and assuming responsibility for Indian health care resulted in confusion and barriers for nurses and patients. This issue not yet resolved. Need evaluation on whether Indigenous populations actually valued the formal healthcare system introduction to their communities.
Rutherdale, M. (2008). Cleansers, cautious caregivers, and optimistic adventurers: A proposed typology of Arctic Canadian nurses	Historical Original Research Paper, Chapter in Scholarly Edited Book	Understand the ways in which the individual personalities of the nurses shaped the nurse-patient dynamic. Discerning nursing types to lend some complexity to the history of northern nursing.	Northern Nurses, Arctic Nurses, Canada, Northern Manitoba and Northwest Territories.	Nurse's approach and effectiveness varied greatly between three different styles of nurses. Cleansers-focused on cleanliness, hygiene, comportment, and appearance. Interventionists. Cautious Caregiver- self-questioning and curious. Culturally and medically reflective and conscious. Optimistic Adventurer- seeking adventure, opportunity to learn about different cultures, people, environments, places.	Suggests it is important to consider individual nursing experiences in the local context in which they occurred to better understand the complexities and nuances of northern nursing.
Quiney, L. (2008). “Suitable young women”: Red Cross nursing pioneers and the crusade for healthy living in Manitoba, 1920–30.	Historical Original Research Paper, Chapter in Scholarly Edited Book	Examines the goals of the state to ensure healthy Canadian Citizenry for the future through the CRCS and the nurses charged with carrying out this mission.	Manitoba, Nurses, Canadian Red Cross Society (CRCS), Rural and Remote	CRCS spread “civilization” to rural and remote areas, especially in western Canada. Help develop citizenship and collective welfare. Provincial support of existing training programs was used to increase the supply of nurses. Standards were set for university programs, supporting the professionalization of nursing. Nurses experienced long hours, heavy workloads, extensive travel, generalized and multidisciplinary scope.	University accreditation of public health nurses had the potential to raise the status of nursing in MB, however there was a lack of programs in MB and nurses had to complete their training out of province. Organization expected cooperative funding systems from rural centers, develop collective welfare. Later realized self-sufficiency would not be possible in most communities due to demographic, economic, and environmental circumstances.
Elliott, J. (2008). (Re)constructing the identity of a Red Cross outpost nurse: The letters of Louise de Kiriline, 1927–36.	Historical Original Research Paper, Chapter in Scholarly Edited Book	Reconstruct Identity of a Red Cross Outpost Nurse. Attention given to identities of the individual outside of nursing.	Outpost Nursing, Ontario (with broader Canadian/ Northern relevance), Canada	Identity of outpost nurses as “modest foot soldiers in crusade for good health” or “angels of the north”. This identity helped to legitimize working women in male dominated positions and promote the image and reputation of CRCS. Enduring professional, gendered, and class stereotyping of rural nursing. 85% of division's outpost nurses were young, single, Canadian born women Canada had paternalistic medical model compared to Sweden. Gender, race, class, and region made individuals social identity.	Suggested qualities of an outpost nurse: independent, confident, like challenges and adventure, desire for autonomy. Incentives for rural outpost nurses: subsidized accommodation, domestic help, freedom of travel and dress (more than normally allowed in twentieth century feminine standards), access to cars, and liveable wage. Individuals who did not fit gender, race, class, and region ideals risk social isolation and rejection.
Rutherdale, M. (2010). Nursing in the North and writing for the South: The work and travels of Amy Wilson.	Historical Original Research Paper, Chapter in Scholarly Edited Book	Examine the text of Amy V. Wilson to determine what her writing can describe about nursing and northern healthcare, Native/newcomer relations, gender dynamics in northern Canada, and the act of memorializing a life in autobiographical form.	Northern Canada, Outpost Nursing	Public health nursing in rural areas was not highly desired work. Many rural prairie women went into nursing for incentives including free room and board, urban lifestyle, opportunities to make professional urban contacts, and acquire highly portable skills. Rural nurses were portrayed like “missionaries of the Canadian state”. Most Northern nurses were intrusive, disruptive, and judgemental or suspicious of Aboriginal practices. Experienced isolation and independence as the sole provider in the community.	The north was portrayed as land for hardy white men, and Northern nurses were praised for surviving the North, yet Indigenous women have thrived there for years. Wilson had an open-minded attitude towards Indigenous medicine and willing to sacrifice southern comforts to achieve her professional goals and desire to live and travel in the North.
Drees, L. M. (2010). Training Aboriginal nurses: The Indian Health Services in Northwestern Canada, 1939–75	Historical Original Research Paper, Chapter in Scholarly Edited Book	Contribute a preliminary description of Indigenous involvement in public health care delivery and the context of that work.	Indigenous Healthcare Workers, Canada	Nursing stations were viewed as the foundation of IHS system. Most medical and nursing staff were not local and non-Indigenous, most support staff were local and Aboriginal. Centralized hierarchical hiring practices. The Department of Native Health and Welfare recruited and trained Indigenous patients to work in the facilities they attended to create Indigenous workforce in healthcare. Common for Aboriginal women to seek nurse training in USA as Canadian schools did not allow non-white individuals to enroll.	After WWII federal gov. Incentives to recruit Indigenous employees to IHS, primarily focused on aids and helpers rather than fully certified nurses. Piecemeal efforts of the Canadian state to involve Indigenous peoples as health care workers reveal its lack of commitment to devolving the responsibility for formal healthcare to Indigenous communities in this period. Devolution did not occur until the 1980s.
Elliot, J. (2010). A Negotiated process: Outpost nursing under the red cross in Ontario, 1922–84.	Historical Original Research Paper, Chapter in Scholarly Edited Book	Explore how issues of gender, class, professionalism and authority have the potential to expand, sustain, or constrain both nurses’ work and personal activities, helping to illustrate that outpost nursing with the CRCS was often a negotiated process.	Ontario, Canadian Red Cross Society (linked to Western Canada), Canada, Outpost Nursing	Incentives for outpost nursing include a degree of independence (practice, personal, financial), marketable skills and education, travel, adventure, status associated with being a nurse. Stable wage, own home. Nursing supervisors retained power and authority of the nurses’ postings. Supervisors linked outposts to the Toronto office and hospital boards. Nurses experienced high moral scrutiny, limited resources, extreme poverty. Professional autonomy bound by the traditional hierarchy of power between medicine and nursing.	Nursing services were accepted more readily in Indigenous communities where nurses were sensitive to cultural differences and were able to develop respectful relationships with locals. What nurses were taught did not reflect the reality of outpost nursing. “Nurses’ work had to be continuously negotiated and renegotiated within parameters by contexts of gender, race, class, which shaped both ideologies and material practices of employers, community members, and physician colleagues, as well as those of nurses themselves”.
McBain, L. (2010). Caring, curing, and socialization: The ambiguities of nursing in Northern Saskatchewan, 1944–57.	Historical Original Research Paper, Chapter in Scholarly Edited Book	Examine the role of nurses as harbingers of social change in Northern Saskatchewan between 1944 and 1957.	Northern Saskatchewan, Canada, Indigenous	Northern healthcare based on humanitarian need, genuine concern for welfare, and the state need to assimilate Indigenous population. Inappropriate programs and inadequate support hindered modernization of the north, resulting in colonial relationships, under resourcing, and marginalization. Nurses stated loneliness, isolation, and subsequent depression as reasons for leaving. Socialization is limited by cultural, class, and gender boundaries. Submissive to the directions of nursing directors and medical health officers.	Suggests colonial relationship between northern and southern SK continues to exist. Sedentary communities were a new way of lifestyle for Indigenous communities after WWII. Was no consultation with North before introducing the PH nursing model. “In all likelihood government did not view nursing as a means to advance their agenda, because if they had, the nursing posts would have been better supported, rather than chronically underfunded”.
