# Supplementary material for: Help Wanted, Experience Preferred, Stamina a Must: A Narrative Review of the Contextual Factors Influencing Nursing Recruitment and Retention in Rural and Remote Western Canada from the Early Twentieth Century to 2023
Source: Can J Nurs Res. 2023 Oct 6;56(2):134–50. doi: 10.1177/08445621231204962 (PMC11032004; doi:10.1177/08445621231204962)
Supplement: sj-docx-1-cjn-10.1177_08445621231204962 - Supplemental material for Help Wanted, Experience Preferred, Stamina a Must: A Narrative Review of the Contextual Factors Influencing Nursing Recruitment and Retention in Rural and Remote Western Canada from the Early Twentieth Century to 2023 [file sj-docx-1-cjn-10.1177_08445621231204962.docx]

Literature Search Terms

The search terms included were “registered nurse OR nurse OR outpost nurse” AND “recruitment and retention OR intent to leave OR personnel shortages OR nursing mobility” AND “rural nursing OR remote nursing OR Northern nursing OR rural health services OR outpost nursing” AND “Canada OR Western Canada OR Manitoba OR Saskatchewan OR Alberta OR British Columbia OR Northern Canada”. All searches were expanded to include equivalent terms. Repeat searches were done to expand the results for historical content by adding the terms “graduate nurse” and “twentieth-century nursing OR interwar period”.
